# Advancements in the treatment of metastatic hormone-sensitive prostate cancer

**DOI:** 10.3389/fonc.2022.913438

**Published:** 2022-08-18

**Authors:** Hengping Li, Mao Zhang, Xiangrong Wang, Yang Liu, Xuanpeng Li

**Affiliations:** Department of Urology, Gansu Provincial Hospital, Lanzhou, China

**Keywords:** prostate cancer, novel hormone therapy, chemotherapy, ADT, mHSPC

## Abstract

In the last decade, there have been substantial improvements in the outcome of the management of metastatic hormone-sensitive prostate cancer (mHSPC) following the development of several novel agents as well as by combining several therapeutic strategies. Although the overall survival (OS) of mHSPC is shown to improve with intense androgen deprivation therapy (ADT), combined with docetaxel, as well as other novel hormonal therapy agents, or alongside local intervention to the primary neoplasm. Notably, luteinizing hormone-releasing hormone (LHRH) antagonists are known to cause fewer cardiovascular side effects compared with LHRH agonists. Thus, in this mini review, we explore the different approaches in the management of mHSPC, with the aim that we may provide useful information for both basic scientists and clinicians when managing relevant clinical situations.

## Introduction

Prostate cancer (PCa) is one of the most common cancers of the urino-genital system; its associated morbidity has progressively increased in the last decade (1). The morbidity and mortality were approximately 1.4 million and 375,000, respectively, in 2020 (2). The incidence of PCa in China has also significantly increased, accounting for 34.2% of the total PCa in Asia (3, 4). Metastatic hormone-sensitive prostate cancer (mHSPC) is responsive to androgen deprivation therapy (ADT) with overall survival (OS) of 42 months following ADT treatment (5). To improve the OS and quality of life (QoL) of mHSPC patients, many novel approaches to the management of mHSPC have been identified in the last decade. Our review aims to outline the advances in the treatment of mHSPC.

## Androgen deprivation therapy

Recent advances in ADT drug therapy predominantly relate to the manufacturing of luteinizing hormone-releasing hormone (LHRH) antagonists, such as degarelix and relugolix.

Degarelix is a luteinizing hormone-releasing hormone (LHRH) antagonist for castration and testosterone suppression administered *via* a subcutaneous injection. In a randomized, parallel-group, phase III clinical study, Klotz et al. reported that testosterone suppression (≤0.5 ng/ml) was achieved in 97.2%, 98.3%, and 96.4% of the intention-to-treat population in degarelix 240/80 mg, degarelix 240/160 mg, and leuprolide groups, respectively. On Day 3, testosterone suppression was achieved in 96.1% and 95.5% of these patients, with a median testosterone (0.24–0.26) response to the degarelix 240/80 mg and 240/160 mg groups, respectively. Moreover, testosterone suppression was increased by 65% in the leuprolide group (6). These data suggest that degarelix is similar to leuprolide in achieving castration level (6). Compared to the 3-month formulation of goserelin, the 1-month formulation of degarelix has a limited clinical application (7). Another phase III study explored formulations with more convenient clinical applications and reported that the cumulative castration rate was 95.1% in the degarelix group and 100.0% in the goserelin group. This indicated that the 3-month formulation of degarelix was not inferior to goserelin in relation to testosterone suppression; degarelix decreased the testosterone level to a castration level on Day 3, while testosterone surged by 52.74% in the goserelin group and did not reach the castration level until Day 28 (8).

Relugolix is an oral LHRH antagonist. A multinational, randomized, phase III study showed that castration was maintained in 96.7% of the patients in the relugolix group compared with 88.8% in the leuprolide group. This indicated that relugolix was superior to leuprolide in all endpoints (all p < 0.001). The major cardiovascular adverse effects were reported by 2.9% of the patients in the relugolix group vs 6.2% of those in the leuprolide group, indicating that relugolix was superior to leuprolide in relation to sustained testosterone suppression with lower cardiovascular adverse effects (7).

### Comparison of luteinizing hormone-releasing hormone antagonists and agonists

A phase II study investigated the impact of LHRH antagonists on cardiovascular disorders (CVDs) and reported that major cardiovascular and cerebrovascular events developed in 20% of patients in the LHRH agonist group vs only 3% of those in the LHRH antagonist group (p = 0.013); the absolute risk reduction in cardiovascular-related events in the antagonist group was 18.1% (9). These results suggest that the choice of using an antagonist or agonist may in PCa patients, with preexisting CVD, may differentially affect CVD (9). To provide more evidence, four eligible studies (n = 2,059) were discussed in a recent systematic review and network meta-analysis of antagonists and agonists, which demonstrated that compared to agonists, the relugolix and degarelix antagonists showed no significant difference in relation to the 12-month castration rate and that relugolix was ranked first in maintaining castration, suggesting that the two antagonists have similar efficacies but that the antagonists induced less cardiovascular events than the agonists (10). Although no head-to-head comparative study of the two LHRH antagonists has been conducted, degarelix injection is associated with a higher rate of injection-site reactions (40%) and is more difficult to administer, whereas oral relugolix is convenient for patients (7). Thus, these results suggest that LHRH antagonists may be the most efficacious drugs for ADT in the future and that relugolix is more suitable for purposes of clinical application because of its oral route of administration, daily.

## Novel hormonal therapy drugs

Existing Novel hormonal therapy (NHT) drugs for treating mHSPC include abiraterone, enzalutamide, apalutamide, and darolutamide. ADT, combined with some of the aforementioned NHT drugs, is approved for the treatment of mHSPC as recommended by the National Comprehensive Cancer Network (NCCN), American Urological Association (AUA), and European Association of Urology (EAU) guidelines (11–13).

Abiraterone is an inhibitor of 17 alpha-hydroxylase/C17, 20-lyase (CYP17), which is produced during androgen synthesis. To manage adverse effects related to mineralocorticoid excess, such as hypokalemia, hypertension, and fluid retention, which can occur as a result of CYP17 inhibition, the administration of abiraterone with prednisone or prednisolone at a low dose of 5 mg twice daily is necessary (14). The LATITUDE trial reported that the median OS was significantly prolonged in the abiraterone+ADT group compared with the placebo+ADT group [NA vs 34.7 months, hazard ratio (HR): 0.62, 95% CI 0.51–0.76; p < 0.001] and that abiraterone significantly benefitted the median radiographic progression-free survival (rPFS) (33 vs 14.8 months, HR: 0.47, 95% CI 0.39–0.55; p < 0.001) in mHSPC; the final follow-up results showed that the median OS was significantly prolonged in the abiraterone group (53.3 vs 36.5 months, HR: 0.66, 95% CI 0.56–0.78; p < 0.0001) (15–17), which is consistent with the findings of the STAMPEDE study (18). The LATITUDE study also reported that the median OS of patients with the high-volume disease was 49.7 months in the abiraterone group and 33.3 months in the placebo group (HR: 0.62, 95% CI 0.52−0.74; p < 0.0001) but that the median OS showed no significant difference in the patients with the low-volume disease (16). The *post-hoc* analysis of the LATITUDE trial showed that the median time to prostate-specific antigen (PSA) progression was 33.2 months in the abiraterone group and 7.4 months in the placebo group (HR: 0.3; p < 0.001). Moreover, a significantly higher PSA_50/90_ was achieved in the abiraterone group than in the placebo group (RR: 1.36/2.30; p < 0.001), and the risk of death was significantly reduced in patients who had a PSA_50/90_ response compared with patients who did not have a PSA response (19). A *post-hoc* exploratory analysis of the LATITUDE trial also suggested that abiraterone treatment improved both rPFS and OS in men with mHSPC and visceral disease, especially those with lung metastases, and that men with liver metastases had a poorer prognosis (20). A *post-hoc* subgroup analysis was performed on the STAMPEDE study, in which the mHSPC patients in the STAMPEDE study underwent stratification using the LATITUDE risk criteria/the CHAARTED volume criteria revealed different outcomes showing that the OS and failure-free survival (FFS) were significantly prolonged by abiraterone compared with the placebo in the low-risk group (HR: 0.66, 95% CI 0.44–0.98, HR: 0.24, 95% CI 0.17–0.33). Also, the same conclusion was drawn in the other subgroups. No significant difference was found in the OS or FFS between the high-risk and low-risk groups, but the number of patients retreated in the low-risk group was fourfold higher than that in the high-risk group (21). The STAMPEDE and LATITUDE studies revealed conflicting conclusions on mHSPC with low-volume disease, which may be associated with the characteristics of the enrolled patients or the number of patients in the low-risk group.

Enzalutamide is a pharmaceutical that blocks an androgen receptor (AR) activity at three levels: 1) AR nuclear translocation, 2) DNA binding, and 3) coactivator recruitment. The ENZAMET study estimated a 3-year OS using the Kaplan–Meier estimator and reported that the 3-year OS was 80% in the enzalutamide+ADT group (based on 94 events) and 72% in the first-generation anti-androgen+ADT group (based on 130 events) in mHSPC patients; enzalutamide also significantly benefitted secondary endpoints, but a high incidence of lassitude, epilepsy, or other adverse effects was observed in the enzalutamide group (22). The ARCHES study also showed that compared with the placebo, enzalutamide significantly reduced the risks of death (HR: 0.39, 95% CI 0.30–0.50; p < 0.001), as well as a first symptomatic skeletal event, castration resistance, and pain progression (23). *Post-hoc* analysis of the ARCHES study further clarified that compared to the placebo, enzalutamide reduced the risks of radiographic progression of bone metastases (HR: 0.33, 95% CI 0.22–0.49) and bone metastases with lymphatic metastasis (HR: 0.31, 95% CI 0.21–0.47) but did not significantly reduce the risk of lymph node metastases (24). The analysis of health-related quality of life (HRQoL) showed that enzalutamide maintained a high QoL and a low symptom burden in mHSPC patients (25). In brief, enzalutamide has clinical benefits for all mHSPC patients who have or have not received local or systemic therapy, regardless of disease burden and risk (26).

Apalutamide is an anti-androgen drug similar to enzalutamide, but it has a higher affinity to AR (23). The TITAN study showed that at 24 months, the apalutamide+ADT group, as well as the placebo+ADT group, had an rPFS of 68.2% vs 47.5% (HR: 0.48, 95% CI 0.39–0.60; p < 0.001) and an OS of 82.4% vs 73.5%, respectively (HR: 0.67, 95% CI 0.51–0.89; p = 0.005), which suggests that rPFS was significantly prolonged in the apalutamide group compared with the placebo group (27). Subgroup analysis showed that the time to pain progression was significantly prolonged in the apalutamide group compared to the placebo group (p < 0.0146), but no significant difference was noted regarding the incidence of lassitude between the two groups (28). As the treatment of PCa has racial disparities, the therapeutic results of the East Asian populations in the TITAN study were analyzed, which demonstrated consistent results with participants involved worldwide. The only inconsistency was that the main adverse effect was a rash (29, 30). According to the final OS results in the TITAN study, apalutamide reduced the risk of death by 35% before crossover (HR: 0.65, 95% CI 0.53–0.79; p < 0.0001) vs 48% after crossover in 208 patients (HR: 0.52, 95% CI 0.42–0.64; p < 0.0001) (31, 32).

Darolutamide is another AR inhibitor. In the latest ARASENS study in mHSPC, the results showed that compared to the placebo+ADT+docetaxel group, the risk of death was significantly reduced by 32.5% in the darolutamide+ADT+docetaxel group (HR: 0.68,95% CI 0.57–0.80; p < 0.001) and that darolutamide was beneficial for all secondary endpoints and subgroups (33). The ARANOTE study is a randomized, double-blinded, placebo-controlled clinical study currently in progress, designed to compare the efficacy of darolutamide+ADT vs ADT alone, in mHSPC treatment.

Although combination therapy with any of the above four NHT drugs provides a significant OS benefit compared with ADT alone, the best therapeutic sequences are still unclear. Regarding adverse effects on the central nervous system (CNS), the available evidence suggests that CNS-related adverse effects are less prevalent with darolutamide than with enzalutamide and apalutamide due to the moderate blood–brain barrier penetration of apalutamide and enzalutamide compared with the lower blood–brain barrier penetration of darolutamide and abiraterone (34, 35).

## Chemotherapy

The GETUG-AFU15 study showed no difference in the median OS between docetaxel and ADT alone in mHSPC (36), but the *post-hoc* analysis suggested that no statistically significant OS benefit was achieved in the high-volume disease in the docetaxel group (37). Subsequently, the CHAARTED study showed that the median OS was prolonged by 13.6 months when using docetaxel compared with using ADT alone (HR: 0.61, 95% CI 0.47–0.80; p < 0.001) in all mHSPC patients. Meanwhile, the median OS was prolonged by 17 months in the subgroup with high-volume disease (HR: 0.60, 95% CI 0.45–0.81; p < 0.001). There was no significant difference in the subgroup with low-volume disease (38). The updated data of the CHAARTED study showed that the median OS was prolonged by 10.4 months in all enrolled patients (HR: 0.72, 95% CI 0.59–0.89; p = 0.0018) and by 16.8 months in patients with high-volume disease in the docetaxel group compared with patients receiving ADT alone (HR: 0.63 95% CI 0.50–0.79; p < 0.001). However, no OS benefit was achieved in the group of patients with a low-volume disease (39). The subgroup analysis of the STAMPEDE study also showed that, compared to the standard of care (SOC) alone, docetaxel+SOC significantly prolonged the median OS in mHSPC patients (81 vs NA HR: 0.78, 95% CI 0.66–0.93; p = 0.006) (40). The long-term follow-up in the STAMPEDE study further indicated that docetaxel was beneficial for the median OS of mHSPC patients with either high or low burden (41). The conclusion of the STAMPEDE study regarding the benefits of docetaxel for patients with low-volume disease contradicts the results of the CHAARTED study, which may be associated with the characteristics of the enrolled patients. The Cochrane review revealed that compared with ADT alone, early docetaxel treatment reduced the risks of death by any cause for mHSPC patients (HR: 0.77, 95% CI 0.68–0.87, I^2^ = 0%) (42). As treatment with docetaxel is beneficial for patients with mHSPC, according to the STAMPEDE study and the Cochrane review, docetaxel is recommended by the NCCN, AUA, and EUA guidelines (11–13).

## Local intervention of the primary neoplasm

Although systemic therapy is important for mHSPC patients, accumulating evidence suggests that for mHSPC patients with low-volume disease, cytoreductive procedures, combined with systemic therapy, such as radiotherapy (RT) to the primary tumor and cytoreductive prostatectomy, can significantly improve the OS. However, such procedures need to be supported by a large number of randomized controlled trials (43). The HORRAD study identified no significant difference in the OS between the RT+ADT and ADT-alone groups, but the time to PSA progression was 15 months in the RT group vs 12 months in the ADT-alone group (HR: 0.78, 95% CI 0.63–0.97; p = 0.02). Subgroup analysis of the HORRAD study suggested that RT tended to be beneficial for HSPC patients with the low-volume disease compared to those with high-volume disease, but the difference was not statistically significant (44). Furthermore, the STAMPEDE study showed that RT significantly improved the FFS (HR: 0.76, 95% CI 0.68–0.84; p < 0.0001) but not the OS in all mHSPC patients; however, RT significantly improved the OS in the low-volume disease group compared to the high-volume disease group (HR: 0.68, 95% CI 0.52–0.90; p = 0.007) (45). In a retrospective analysis, Morgan et al. found that the RT significantly benefitted the median OS (47.7 vs 26.3 months, HR: 0.69, 95% CI 0.50–0.94; p = 0.02) compared to ADT alone, and such benefit was more remarkable in patients who had survived for at least 1 year (52.2 vs 39.8 months, HR: 0.73, 95% CI 0.54–0.98; p = 0.04) (46). The STOPCAP meta-analysis also demonstrated that a 3-year survival benefit was achieved in 7% of patients with less than five bone metastases (HR: 1.47 95% CI 1.11–1.94; p = 0.007), suggesting that RT for prostate should be considered for mHSPC patients with the low-volume disease (47). Cytoreductive prostatectomy is another method for the local interventions of mHSPC. Heidenreich et al. found that cytoreductive prostatectomy+ADT prolonged the PFS by 12.1 months (p = 0.032), increased the disease-specific survival rate by 11.4%, and increased the OS rate by 12.4%, compared with therapy with ADT alone (48). The TRoMbone clinical trial, which was designed to investigate the efficacy of cytoreductive prostatectomy+ADT vs ADT alone for the treatment of mHSPC patients with low-volume disease, is currently in progress (49). Cytoreductive cryotherapy also shows a survival benefit in mHSPC patients with low-volume disease. Sheng et al. reported that cytoreductive cryosurgery+ADT significantly prolonged the PFS compared to ADT alone in mHSPC patients with the low-volume disease (35 vs 25 months; p = 0.0027) (50). In conclusion, based on the above benefits, local interventions of primary neoplasm are recommended for mHSPC with the low-volume disease according to the AUA guidelines (13).

## Comparison of combination therapy

Although NHT drugs and chemotherapy have significant efficacy in the treatment of mHSPC patients, the optimal drugs for the best therapeutic option should be determined. Sathianathen et al. performed a meta-analysis by focusing on papers published from January 2014 up to June 2019 and reported that the combination of ADT+docetaxel/abiraterone/enzalutamide/apalutamide is superior to ADT alone and that enzalutamide+ADT has the lowest absolute risk among all studied combination therapies (HR: 0.53, 95% CI 0.37–0.75) (51). Wang et al. performed a network meta-analysis and reported that the improvement in OS was achieved with the use of (from largest to smallest improvement) abiraterone, apalutamide, and docetaxel (HR: 0.61, 95% CI 0.54–0.70; HR: 0.67 95% CI 0.51–0.89; HR: 0.79 95% CI 0.71–0.89), whereas the improvement in rPFS was achieved with the use of (from largest to smallest improvement) enzalutamide, apalutamide, abiraterone, and docetaxel (HR: 0.39, 95% CI 0.30–0.50; HR: 0.48, 95% CI 0.39–0.60; HR: 0.51, 95% CI 0.45–0.58; HR: 0.67, 95% CI 0.60–0.74). Docetaxel had the largest risk of adverse effects, while abiraterone had a slightly increased risk; however, the other drugs had no significantly increased risk (52). Similarly, Ferro et al. performed a network meta-analysis and suggested that compared to chemotherapy, NHT significantly improved the OS of mHSPC patients (HR: 0.78, 95% CI 0.67–0.91) (53). The volume of disease also affects the prognosis of mHSPC. In a meta-analysis conducted by Sathianathen et al., focusing on papers published from January 2014 up to June 2019, the subgroup analysis revealed that each combination therapy significantly improved OS compared with ADT alone in the high-volume disease group; however, no significant difference was observed between the combination therapies. Enzalutamide combination therapy improved the OS to a great extent compared with the other combination therapies in the low-volume disease group (HR: 0.38, 95% CI 0.20–0.68) (51). Wenzel et al. also performed a network meta-analysis of the systemic treatment for mHSPC patients and reported that abiraterone, apalutamide, and docetaxel prolonged the OS (HR: 0.59, 95% CI 0.50–0.69; HR: 0.68, 95% CI 0.50–0.69; HR: 0.73, 95% CI 0.62–0.85) but that enzalutamide did not prolong the OS compared to ADT. Moreover, the ranking analysis showed that the improvement in the OS was achieved with the use of (from largest to smallest improvement) abiraterone, apalutamide, and docetaxel; however, only abiraterone and enzalutamide prolonged the OS in the high-volume disease mHSPC subgroup (HR: 0.61, 95% CI 0.47–0.79; HR: 0.43, 95% CI 0.26–0.72), while apalutamide and docetaxel did not prolong the OS compared to ADT alone. In addition, the ranking analysis showed that the OS benefit was achieved with the use of (from largest to smallest improvement) enzalutamide and abiraterone in the low-volume disease mHSPC subgroup (54). Thus, the best OS was achieved with abiraterone in high-volume disease mHSPC patients and enzalutamide in low-volume disease mHSPC patients (54).

Many comparative studies on abiraterone and chemotherapy have been conducted. Kassem et al. performed a network meta-analysis to compare abiraterone and docetaxel in the treatment of mHSPC, and they suggested that abiraterone therapy had a better PFS and lower drug toxicity than docetaxel but that there was a trend for the abiraterone therapy to benefit OS without statistical significance (55). In a meta-analysis conducted by Wenzel et al., abiraterone treatment of mHSPC patients with high-volume disease resulted in a median OS of 50.1 months, which exceeded that of docetaxel (45.9 months) and ADT alone (34.0 months); no significant difference in the median OS was identified between docetaxel and ADT alone in the low-volume disease group (54). In a retrospective, multicenter study that compared the efficacy and safety of abiraterone and docetaxel in the treatment of mHSPC, the abiraterone+ADT group had a significantly longer PFS1, PFS2 (PFS1/PFS2, time from start of ADT to clinical, biochemical, or radiographic progression during first/second line or death from any cause) and OS as compared with the docetaxel+ADT group (23 vs 13 months; p < 0.001; 48 vs 33 months; p = 0.006; 80 vs 61 months; p = 0.040); according to a multivariate analysis of PFS1 (HR = 0.34, 95% CI 0.183–0.623; p = 0.001) and PFS2 (HR = 0.33, 95% CI 0.128–0.827, p = 0.018), abiraterone+ADT was significantly better than docetaxel+ADT, but both resulted in similar OS and toxic effects (56). The STAMPEDE clinical study demonstrated that the mean QoL score, over the period of 2 years, was +3.9 points higher in patients treated with abiraterone than in those treated with docetaxel, which fails to meet the predefined criterion for a clinically meaningful difference of >4.0 points; the mean QoL score was +5.7 points higher over 1 year, +7.0 points higher at 12 months, and +8.3 points higher at 24 months, suggesting that the patient-reported QoL was better in patients treated with abiraterone compared with those treated with docetaxel over a 2-year period (57).

## Precision treatment

As gene mutations are significantly associated with the prognosis of metastatic castration-resistant prostate cancer (mCRPC) (58–60), previous studies have also reported the relationship between gene mutation and mHSPC (61–63). Velez et al. retrospectively detected the TP53, PTEN, and RB1 mutations in 97 patients in a single center and identified tumor suppressor gene (TSG) mutations in 48 (49%) patients treated with abiraterone+ADT and in 49 (51%) patients treated with docetaxel+ADT. Velez et al. found that the median PFS was 13.1 months in the TSG-normal group vs 7.8 months in the TSG-altered group (p = 0.005); subgroup analysis showed that the median PFS was lower in TSG-altered patients compared to TSG-normal patients in the abiraterone+ADT group (8.0 months, 95% CI 5.8–13.8; 23.2 months, 95% CI 13.1–NA), but no difference was observed between the docetaxel+ADT subgroups. Using multivariable analysis, Velez et al. reported that altered TSG predicted the prognosis of mHSPC in early first-line treatment (HR: 2.37, 95% CI 1.42–3.96; p < 0.001) and that detection of the TSG mutation was superior to the clinical criteria (61). However, several gene mutations benefit from hormonal therapy. In a retrospective study, Swami et al. investigated PCa patients who received standard ADT only and who were identified with SPOP gene mutations [n = 121 total patients, 25 patients with mutant SPOP (mtSPOP) and 96 patients with wild-type SPOP (wtSPOP)]; the study reported that standard ADT therapy resulted in a longer median PFS and median OS in patients with mtSPOP compared to patients with wtSPOP (35 vs 13 months, HR: 0.47; p = 0.016; 97 vs 69 months, HR: 0.32; p = 0.027) (63).

## Summary and prospect

Revolutionary progress has been made in the development of mHSPC treatment options, notably including LHRH antagonists, NHT drugs, chemotherapy, and local intervention of low-volume disease of mHSPC, as well as in certain combined treatments. This has provided major benefits for mHSPC patients over the last decade. Nonetheless, further research is still needed to determine the optimal combinational therapies of these drugs. Therefore, multicenter prospective studies with larger sample sizes will inevitably be conducted in the future.

## Author contributions

XP-L, YL, XR-W participated in drafting the manuscript. MZ was responsible for revising the manuscript. HP-L designed the study and was responsible for revising the manuscript. All authors contributed to the article and approved the submitted version.

## Funding

This work was supported by a grant from Gansu Provincial Hospital (17GSSY3-4).

## Conflict of interest

The authors declare that the research was conducted in the absence of any commercial or financial relationships that could be construed as a potential conflict of interest.

## Publisher’s note

All claims expressed in this article are solely those of the authors and do not necessarily represent those of their affiliated organizations, or those of the publisher, the editors and the reviewers. Any product that may be evaluated in this article, or claim that may be made by its manufacturer, is not guaranteed or endorsed by the publisher.
